# Triangle of Safety Technique: A New Approach to Laparoscopic Cholecystectomy

**DOI:** 10.1155/2009/476159

**Published:** 2009-06-17

**Authors:** Abdulrahman Faraj Almutairi, Yousef A. M. S. Hussain

**Affiliations:** ^1^Department of Surgery, Farwaniya Hospital, 81002 Farwaniya, Kuwait; ^2^Department of Surgery, Al-Adan Hospital, 47370 Al-Adan, Kuwait

## Abstract

*Backgrounds and Study Aims*. Common bile duct (CBD) injury is one of the most serious complications of laparoscopic cholecystectomy (LC). Misidentification of the CBD during dissection of the Calot's triangle can lead to such injuries. The aim of the authors in this study is to present a new safe triangle of dissection. *Patients and Method*. 501 patients under went LC in the following approach; The cystic artery is identified and mobilized from the gall bladder (GB) medial wall down towards the cystic duct which would simultaneously divide the medial GB peritoneal attachment. This is then followed by dividing the lateral peritoneal attachment. The GB will be unfolded and the borders of the triangle of safety (TST) are achieved: cystic artery medially, cystic duct laterally and the gallbladder wall superiorly. The floor of the triangle is then divided to delineate both cystic duct and artery in an area relatively far from CBD. *Results*. There were little significant immediate or delayed complications. The mean operating time was 68 minutes, nearly equivalent to the conventional method. *Conclusions*. Dissection at TST appears to be a safe procedure which clearly demonstrates the cystic duct and may help to reduce the CBD injuries.

## 1. Introduction

Laparoscopic cholecystectomy has become the standard method of treatment for the removal of a diseased gallbladder. The technique most commonly employed is the infundibular approach which entails dissecting the gallbladder from its neck upward, after dissecting the cystic artery and the cystic duct using laser or electrocautery [1]. However, a significant increase in the incidence of bile duct injury was noted more than that occurring in the era of open cholecystectomy [2] reaching up to 0.5% as reported by David Flum from the University of Washington [3] and Gigot et al. describing the Belgium experience [4]. Injury occurs as a result of misidentification of the ducts or other technical errors. Issues like poor surgical technique, lack of understanding of how injuries occur, surgeon resistance to convert to open surgery, inadequate visualization, inflammation, and aberrant anatomy are key risk factors [5–7]. The purpose of our new technique is to describe structured steps of dissection in a new anatomical triangle relatively away from CBD which forms one boarder in the Calot's triangle thus reducing misidentification issue and other factors leading to ductal injury. We believe that Triangle of Safety Technique (TST) provides better definition of anatomy in a relatively safer area of dissection and so recommend its routine use for laparoscopic cholecystectomy.

## 2. Patients and Methods

501 patients underwent laparoscopic cholecystectomy for gallbladder disease by the author and team in Farwanyia hospital from January 2001 to December 2008.

**Operative Procedure**

**Figure 1:**
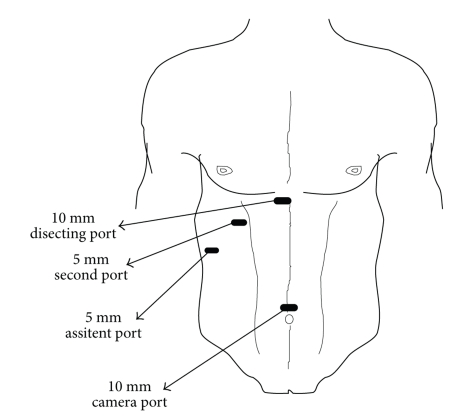
Ports site in LC.

**Figure 2:**
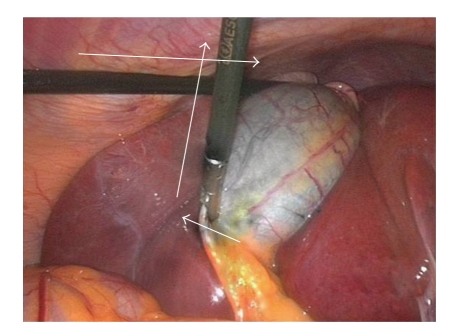
Traction of GB.

The procedure is carried out using the standard four-port technique: the first port is a 10 mm supraumbilical camera port inserted using the open technique method of CO2 insufflation and the other three ports are inserted under direct camera vision (Figure 1). The gallbladder is retracted from the fundus in the flip over manner above the right lobe of the liver by the assistant. The anterior edge of Hartman's pouch is retracted by the surgeon left hand in an outward and lateral direction through the second port thus opening space for establishing the triangle of safety (Figure 2).

The borders of triangle of safety are dissected out in four essential steps using electrocautery hook as follows.

**Figure 3:**
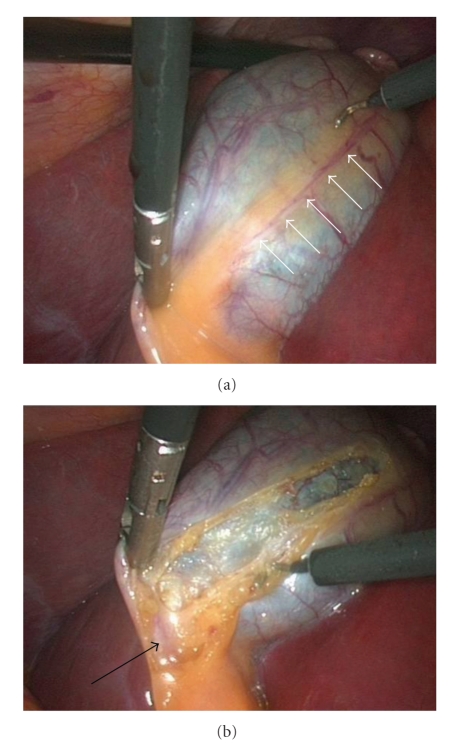
(a) Identifying and dissecting the GB peritoneum above cystic artery (white arrows). (b) Continuing dissection toward cystic duct-infundibular Junction (black arrow).

First step is dissecting the peritoneum over the GB wall in a direction just lateral to and parallel to the cystic artery from mid way along its length down toward the junction of the cystic artery and duct (Figures 3(a), 3(b)). The cystic artery usually follows a constant pathway over the GB wall and in our experience can easily be identified and seen underneath the peritoneum of the GB. Initial difficulty identifying the cystic duct might exist in cases which the peritoneum of the GB is thickened due to inflammation. In these cases the artery can be identified by sweeping the peritoneum to uncover the cystic artery or one of its branches which can then be used to track the artery.

**Figure 4:**
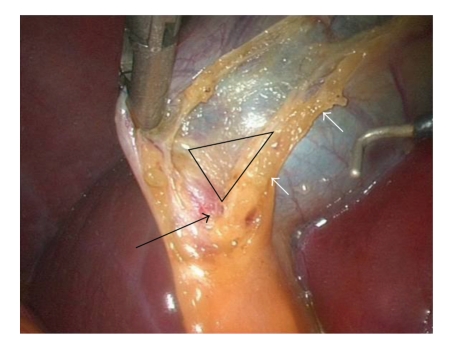
Cystic Artery (white arrows) and junction between cystic duct and artery (black arrow) and the Triangle of Safety.

Second step is dividing the small branches of the cystic artery flaring on the GB wall under layers of peritoneum, one by one, layer by layer, until the dissection reaches a small branch that adheres cystic artery to cystic duct “Calot's artery” [8], forming there junction (Figure 4). Again gentle sweeping of peritoneal covering helps to identify these branches where there is a thick wall GB. This is usually easily done and is facilitated by an edema of the wall of an inflamed GB wall. Further more, any bleeding can easily and safely be controlled by electrocautery as area of dissection is on the GB wall, away from any vital structures. With this step the GB is released from its medial peritoneal attachment allowing the cystic artery to fall down forming the medial border of the triangle of safety (TST) and exposing the other two borders: The posterior wall of the gallbladder and the cystic duct-infundibular junction (Figure 4).

**Figure 5:**
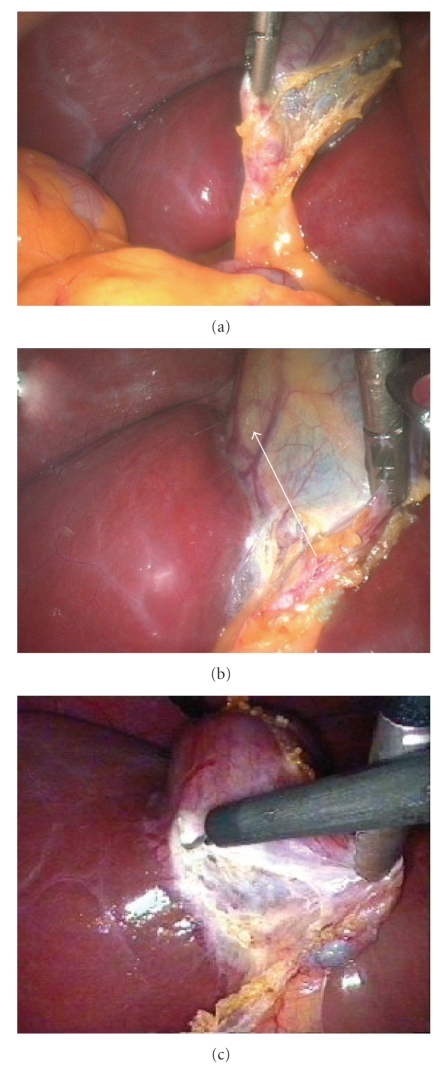
(a) Dividing the lateral peritoneal wall. (b) Dividing the lateral peritoneal wall. (c) Dividing the lateral peritoneal wall.

Third step is releasing the lateral peritoneal attachment (Figures 5(a)–5(c)).

**Figure 6:**
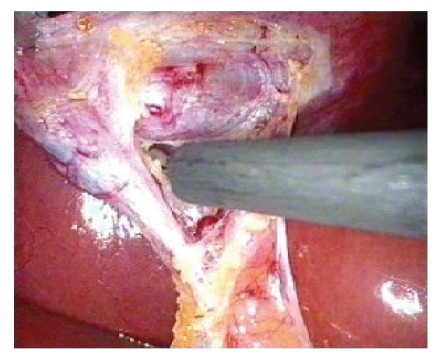
Dividing tissues in Triangle of Safety.

Fourth step is dividing tissues lying among the borders of triangle of safety close to the gallbladder wall reaching the lateral side and avoiding the posterior cystic artery branch (Figure 6).

**Figure 7:**
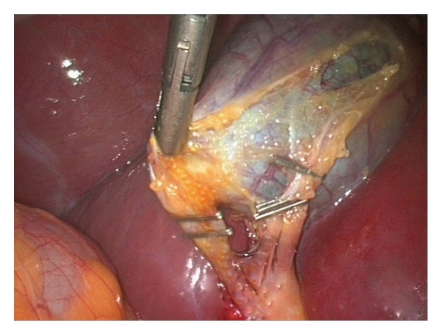
Clipping the cystic artery over the GB wall and the duct in close proximity to the infundibulum.

Finally is clipping and dividing the cystic artery over the GB wall rather in the Calot's triangle will spare dissection and possible injury near the common hepatic duct. This will leave only the cystic duct which can be divided near its junction with the GB infundubulum (Figure 7).

## 3. Results

There were 349 females and 152 males. The mean age was 42 years (range from 14 to 74 years). 80 patients were done as emergencies. The mean operative time was 68 minutes. Patients how underwent conversion to open cholecystectomy before start of dissecting GB due to tense adhesions and nonvisualization of GB were excluded from this study. There was one case converted to open due to bleeding from aberrant cystic artery rising directly from superior mesenteric artery on the lateral side of the GB. GB puncture with bile and stones leak occurred due to vigorous traction rather than electrodithermy. This was considered to be minor complication when compared to injury to the CBD.

## 4. Discussion

Prevention of injury to the ductal system continues to be a matter of considerable concern for any surgeon performing laparoscopic cholecystectomy. An increased incidence of CBD injury has been reported ranging between 0.5% to 3% [9, 10] compared to 0.1%–0.5% [11, 12] in open cholecystectomy.

 Few methods have been advocated to reduce the incidence of ductal injuries which include: routine performance of intraoperative cholangiography [7, 13] and fundus first technique [14, 15]. Many guidelines have been suggested to avoid misidentification of the ducts including instructions for the direction of traction on the gallbladder [16]. In the author's and others opinion all these methods and guidelines are important but still do not emphasize the key issue of misidentification that results in failure to conclusively identify the cystic duct structure before its division. Furthermore way suggested that 97% of CBD injury were due to visual perceptual illusion leading to identifying the CBD as the cystic duct so deliberately cutting it rather than fault in technical skills thus many operative reports describe operation as routine despite missed injuries [5]. Strasberg suggested that no clipping or cutting should be done until the Calot's triangle is cleared from all fat to visulized only tow structures: the cystic artery and duct [17]. However it was left to the surgeon to decide the safest method to reach this critical view without causing injury. We believe adherence to TST by starting the dissection at the GB wall identifying first the cystic artery which will be followed toward the cystic duct-infundibular junction can help in reducing misperception errors because failure to identifying the artery should alert the surgeon toward thinking of anomalies in both the arterial and ductal systems to be more vigilant and careful in his dissection. Other possible advantage to TST is the fact that dividing the peritoneum and braches of cystic duct over GB wall to open the triangle of safety will left the GB infundibulium away from the liver bed uncovering possible short or hidden cystic duct. 

 There are four newly introduced steps in this technique and the remaining steps are carried out in the standard conventional way.

 In TST, dissection starts in an area away from Calot's triangle whereby no ductal or arterial anomalies are encountered.

 Upon reviewing the cystic duct and artery anomalies described in literature, most occur at the level of Calot's triangle [17–20]. TST spares this area. In fact the cystic artery proper and its terminal branches are constant and form a reliable land mark for the initiation of our dissection. Moreover, following the cystic artery branches from the gallbladder wall will clarify if there is a posterior branch which can be preserved to be dissected after the TST view is established.

## 5. Conclusion

TST appears to be a safe technique which clearly demonstrates the anatomy of the cystic duct and reduces misidentification issue and the need for intraoperative cholangiography. As TST dissection occurs at a distance from Calot's triangle, no ductal or arterial anomalies are likely to be encountered, thus minimizing intra- and postoperative complications.
